# Cephalometric evaluation of adult anterior open bite non-extraction treatment with Invisalign

**DOI:** 10.1590/2177-6709.22.5.030-038.oar

**Published:** 2017

**Authors:** Shuka Moshiri, Eustáquio A. Araújo, Julie F. McCray, Guilherme Thiesen, Ki Beom Kim

**Affiliations:** 1 Saint Louis University, Department of Orthodontics (Saint Louis, USA).

**Keywords:** Open bite, Orthodontics, Orthodontic appliances

## Abstract

**Objective:**

The purpose of this study was to evaluate, by means of cephalometric appraisal, the vertical effects of non-extraction treatment of adult anterior open bite with clear aligners (Invisalign system, Align Technology, Santa Clara, CA, USA).

**Methods:**

Lateral cephalograms of 30 adult patients with anterior open bite treated using Invisalign (22 females, 8 males; mean age at start of treatment: 28 years and 10 months; mean anterior open bite at start of treatment: 1.8 mm) were analyzed. Pre- and post-treatment cephalograms were traced to compare the following vertical measurements: SN to maxillary occlusal plane (SN-MxOP), SN to mandibular occlusal plane (SN-MnOP), mandibular plane to mandibular occlusal plane (MP-MnOP), SN to mandibular plane (SN-MP), SN to palatal plane (SN-PP), SN to gonion-gnathion plane (SN-GoGn), upper 1 tip to palatal plane (U1-PP), lower 1 tip to mandibular plane (L1-MP), mesiobuccal cusp of upper 6 to palatal plane (U6-PP), mesiobuccal cusp of lower 6 to mandibular plane (L6-MP), lower anterior facial height (LAFH), and overbite (OB). Paired *t*-tests and descriptive statistics were utilized to analyze the data and assess any significant changes resulting from treatment.

**Results:**

Statistically significant differences were found in overall treatment changes for SN-MxOP, SN-MnOP, MP-MnOP, SN-MP, SN-GoGn, L1-MP, L6-MP, LAFH, and OB.

**Conclusions:**

The Invisalign system is a viable therapeutic modality for non-extraction treatment of adult anterior mild open bites. Bite closure was mainly achieved by a combination of counterclockwise rotation of the mandibular plane, lower molar intrusion and lower incisor extrusion.

## RESUMO

**Objetivo:**

o objetivo desse estudo foi realizar uma avaliação cefalométrica dos efeitos verticais do tratamento sem extração de mordidas abertas anteriores em adultos com o uso de alinhadores (sistema Invisalign, Align Technology, Santa Clara, CA, EUA).

**Métodos:**

foram analisados cefalogramas laterais de 30 pacientes adultos com mordida aberta anterior, tratados com o sistema Invisalign (22 do sexo feminino, 8 do sexo masculino, com idade média ao início do tratamento de 28 anos e 10 meses, e mordida aberta anterior média igual a 1,8mm). Os cefalogramas pré- e pós-tratamento foram traçados e as seguintes medidas verticais foram avaliadas: SN ao plano oclusal maxilar (SN-MxOP), SN ao plano oclusal mandibular (SN-MnOP), plano mandibular ao plano oclusal mandibular (MP-MnOP), SN ao plano mandibular (SN-MP), SN ao plano palatino (SN-PP), SN ao plano gônio-gnátio (SN-GoGn), incisivo central superior ao plano palatino (U1-PP), incisivo central inferior ao plano mandibular (L1-MP), cúspide mesiovestibular do molar superior ao plano palatino (U6-PP), cúspide mesiovestibular do molar inferior ao plano mandibular (L6-MP), altura facial anterior inferior (AFAI) e sobremordida (OB). Testes *t* pareados e estatística descritiva foram utilizados para analisar os dados e as alterações significativas resultantes do tratamento.

**Resultados:**

foram encontradas diferenças estatisticamente significativas durante o tratamento para SN-MxOP, SN-MnOP, MP-MnOP, SN-MP, SN-GoGn, L1-MP, L6-MP, AFAI e OB.

**Conclusões:**

o sistema Invisalign é uma modalidade terapêutica viável para o tratamento sem extração de mordidas abertas anteriores em pacientes adultos. O fechamento da mordida foi obtido principalmente por uma combinação de rotação do plano mandibular no sentido anti-horário, intrusão molar inferior e extrusão do incisivo inferior.

## INTRODUCTION

Open bites pose as one of the more challenging dentofacial deformities in the orthodontic world, as they tend to defy treatment.^1^ ^-^ ^3^ Indeed, many researchers contend that vertical discrepancies are more difficult to manage than those in the anteroposterior dimension.^4^ ^,^ ^5^ The complexity of this particular bite stems from both the mechanics needed to treat it and the efforts to combat its high relapse tendency. Due to lack of anterior contact, anterior open bites can lead to excessive wear of the posterior dentition, as the patient lacks anterior disclusion. Impairments with mastication and speech, in addition to dissatisfaction with the esthetics of an open bite, can negatively impact patients on a psychological and emotional level.^6^

The etiology of anterior open bites is complex and multifaceted. It may develop from either oral habits, excessive growth of lymphatic tissues, tongue position, or a genetic predisposition. While growing patients may be treated with interceptive orthodontic appliances, treatment of adult patients presents a more complex picture once growth has ceased and habit-related sequela assume permanence.^7^ ^,^ ^8^

A meta-analysis on the long-term stability of treatment of anterior open bites found that both surgical and non-surgical correction had success rates greater than 75% (with an 82% mean stability value for patients surgically treated and 75% for patients treated only with orthodontics).^9^ This indicates that nonsurgical orthodontics has nearly equal long-term stability outcomes, while being a less invasive and more economical option for the patient. Non-surgical adult treatment of anterior open bites involves either extrusion of the anterior segment^10^ or, less commonly, intrusion of over-extruded posterior segments.^10^ ^-^ ^12^ The rising popularity of adult orthodontics, and lack of guaranteed stability with both fixed appliance therapy and surgery, has generated impetus to discover more effective treatment modalities for anterior open bites. One alternative practitioners have turned towards is that of clear aligner therapy. 

Upon arrival to the market, Invisalign was promoted as an esthetic alternative to fixed appliances.^13^ Initially, it was indicated for low complexity cases, without skeletal discrepancies, mainly involving mild crowding. Since its inception, the appliance has undergone several alterations to improve its ability to achieve proper alignment and occlusion. The Invisalign system has rapidly evolved and incorporated features ostensibly have enabled it to treat more complex malocclusions. 

Although the literature examining orthodontic treatment with Invisalign is limited, a few investigators have demonstrated its successful management of mild anterior open bites.^14^ ^-^ ^16^ The appliance is purported to have a bite block effect and to maintain vertical control, two traits that make it a possible treatment alternative for open bite cases. Unfortunately, the few published studies are case reports that do not adequately evaluate the appliance’s capacity to maintain vertical control, a parameter that is often worsened by the extrusive effects of fixed appliance therapy. 

Thus, the purpose of this study was to evaluate the vertical effects of non-extraction, adult anterior open bite treatment with the Invisalign system. It would be beneficial to understand Invisalign’s influence on this dimension in order to understand the appliance’s capacity for vertical control.

## MATERIAL AND METHODS

### Sample

Institutional review board approval was obtained before the study (protocol #25918). Initially, the sample size calculation was made with Epi Info^®^ 7 software (CDC, Atlanta, GA, USA), using the following parameters^2^: an average and standard deviation for the vertical position of the lower incisor at pre-treatment of 38.26 ± 2.93 mm and at post-treatment of 40.97 ± 2.74 mm. Using a 90% power and 5% significance level, a sample size of 30 subjects would be sufficient.

Pre-treatment (T_1_) and post-treatment (T_2_) lateral cephalograms of thirty adult anterior mild open bite patients treated with Invisalign were retrospectively collected from three orthodontic private practices. Anterior open bite was defined as a lack of vertical overlap between the upper and lower incisors. The sample was comprised of 22 females and 8 males, with a mean age of 28.81 years (range: 16y 11m to 54y 3m) at the outset of treatment. No discrimination as to Angle classification of malocclusion was made: the sample consisted of 24 Angle Class I patients and 6 Angle Class II patients. No patient presented crowding exceeding 6 mm either in the maxillary or in the mandibular dental arch. Twenty-four sets of records were obtained from practice A, four sets of records were obtained from practice B, and two sets of records were obtained from practice C. These patients were randomly selected between anterior open bite cases that were finished between 2011 and 2015.

The patient selection criteria were as follows:

» Patients were all non-growing at the outset of treatment, determined via the cervical vertebral maturation technique.
» No vertical overlap between the upper and lower incisors, with edge-to-edge canines deemed acceptable.
» No extractions of permanent teeth were performed during treatment.
» No orthognathic surgery was performed as a part of treatment.
» The patients were treated exclusively with Invisalign and anteroposterior elastics during treatment, if necessary.
» Pre- and post-treatment lateral cephalograms were available for each patient.

Care was taken to ensure that all private practitioners had at least elite provider status, indicating that they treat, at minimum, up to one hundred Invisalign cases per year. 

The overall goal of each treatment was to achieve overbite reduction in order to attain vertical overlap, or positive overbite, of the maxillary and mandibular incisors. 

### Data collection

**Figure 1:**
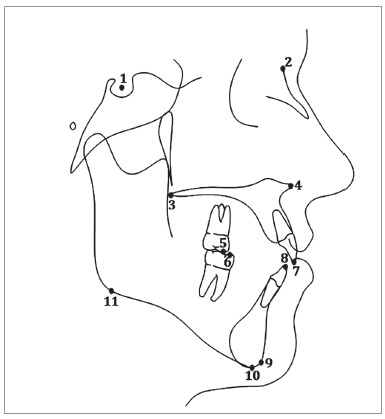
Anatomical landmarks: 1) Sella; 2) Nasion; 3) Posterior nasal spine; 4) Anterior nasal spine; 5) U6 mesiobuccal cusp; 6) L6 mesiobuccal cusp; 7) U1 incisor tip; 8) L1 incisor tip; 9) Gnathion; 10) Menton; 11) Inferior gonion.

**Figure 2:**
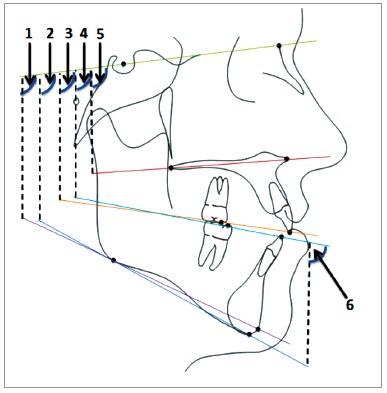
Cephalometric angular measurements: 1) SN-GoGn; 2) SN-MP; 3) SN-MxOP; 4) SN-MnOP; 5) SN-PP; 6) MP-MnOP.

**Figure 3:**
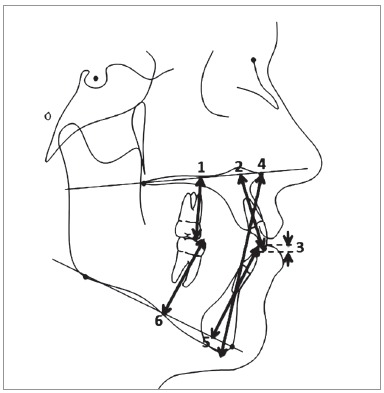
Cephalometric linear measurements: 1) U6-PP; 2) U1-PP; 3) OB; 4) LAFH; 5) L1-GoGn; 6) L6-GoGn.

Pre-treatment and post-treatment digital and analog lateral cephalograms were collected, scanned, and traced for each patient digitally in the Dolphin Imaging 11.8 Premium software (Dolphin Imaging Systems LLC, Chatsworth, CA, USA). Since the lateral cephalograms were obtained from distinct practices, the magnification rate was corrected using this software. Thirty-three hard tissue landmarks were identified and traced, in addition to two reference landmarks on each radiograph (Fig 1). The mandibular and maxillary occlusal planes were manually traced and all occlusal plane measurements were manually measured. Six linear and six angular measurements were performed (Table 1, Figs 2 and 3). 

**Table 1:** Measurements definitions.

Measurement	Definition
SN-MxOP	Angle formed by SN and the maxillary occlusal plane (plane drawn through the mesiobuccal cusp tip of the maxillary molar to the upper central incisor tip)
SN-MnOP	Angle formed by SN and the mandibular occlusal plane (plane drawn through the mesiobuccal cusp tip of the mandibular molar to the lower central incisor tip)
MP-MnOP	Angle formed by mandibular plane (inferior gonion to menton) and the mandibular occlusal plane
SN-PP	Angle formed by SN to palatal plane (ANS to PNS)
SN-MP	Angle formed by SN to mandibular plane (inferior gonion to menton)
SN-GoGn	Angle formed by SN and the plane drawn through the points inferior gonion and gnathion
U1-PP	The millimetric distance between U1 tip and the palatal plane (ANS to PNS)
L1-GoGn	The millimetric distance between L1 tip and the plane drawn through inferior gonion-gnathion
U6-PP	The millimetric distance between the mesiobuccal cusp tip of the maxillary molar and the palatal plane (ANS to PNS)
L6-GoGn	The millimetric distance between the mesiobuccal cusp tip of the mandibular molar and the plane drawn through inferior gonion-gnathion
LAFH	The millimetric distance between ANS and menton
OB	The vertical millimetric distance from U1 tip to L1 tip

### Statistical methods

The data in this study was analyzed via IBM SPSS Statistics 23.0 statistical analysis software (SPSS Inc., Chicago, IL, USA). Descriptive statistics and paired *t*-tests were used to compare the changes between pre- and post-treatment measurements. All digital and manual tracings were performed by the same investigator. To evaluate intra-examiner reliability, 20% of the cephalograms were chosen at random and re-traced; Cronbach’s alpha test was used to determine measurement reliability. Intra-class correlation values of at least 0.80 were considered acceptable in terms of reliability. 

## RESULTS

Average treatment time was 21 months (ranging from 11 to 34 months). Descriptive statistics (Table 2) and paired *t*-tests (Table 3) used to analyze the data revealed that nine of the twelve variables measured were statistically significant in overall treatment change. Statistically significant (*p*< 0.01) changes were found in SN-MxOP, SN-MnOP, MP-MnOP, SN-MP, SN-GoGn, LAFH, overbite, and L1-MP. Statistically significant (*p*< 0.05) changes were also observed in L6-MP. But SN-PP, U1-PP, and U6-PP did not undergo any statistically significant changes. 

**Table 2:** Descriptive statistics at T_1_ and T_2_.

Measurement	Pre-treatment (T_1_)		Post-treatment (T_2_)	
	Mean	SD	Mean	SD
SN-MxOP (degrees)	18.0	5.0	20.6	5.4
SN-MnOP (degrees)	20.7	5.5	16.2	5.6
MP-MnOP (degrees)	20.2	5.1	24.7	4.6
SN-PP (degrees)	7.8	4.2	7.5	4.7
SN-MP (degrees)	40.8	7.2	39.9	6.9
SN-GoGn (degrees)	37.6	7.1	36.7	6.9
LAFH (mm)	74.3	5.3	72.8	5.2
OB (mm)	-1.8	1.2	1.5	0.9
U1-PP (mm)	30.7	2.8	31.2	2.6
L1-MP (mm)	38.3	2.8	39.1	3.1
U6-PP (mm)	25.4	2.2	25.0	2.3
L6-MP (mm)	31.3	2.5	30.7	2.4

**Table 3:** Treatment changes.

	T_1_-T_2_ difference		
Measurement	Mean	SD	Significance
SN-MxOP (degrees)	2.6**	2.4	<0.001
SN-MnOP (degrees)	-4.6**	4.2	<0.001
MP-MnOP (degrees)	4.5**	3.7	<0.001
SN-PP (degrees)	-0.3	2.4	0.505
SN-MP (degrees)	-0.9**	1.5	0.002
SN-GoGn (degrees)	-0.9**	1.6	0.006
LAFH (mm)	-1.5**	2.8	0.006
OB (mm)	3.4**	1.4	<0.001
U1-PP (mm)	0.5	2.0	0.137
L1-MP (mm)	0.8**	1.2	<0.001
U6-PP (mm)	-0.4	1.4	0.118
L6-MP (mm)	-0.6*	1.4	0.022

* denotes changes are significant at *p* < 0.05.

** denotes changes are significant at *p* < 0.01.

Chronbach’s alpha tests for intra-examiner reliability was above 0.80 for all variables except for overbite (Cronbach’s alpha= 0.63). With regard to accuracy of measurements, overall, the original and repeated measurements were at an adequate level of reliability (Table 4). 

**Table 4:** Reliability statistics.

Variable	Cronbach’s alpha
SN-MxOP	0.958
SN-MnOP	0.864
MP-MnOP	0.965
SN-PP	0.950
SN-MP	0.983
SN-GoGn	0.987
LAFH	0.993
OB	0.629
U1-PP	0.837
L1-MP	0.923
U6-PP	0.946
L6-MP	0.825

## DISCUSSION

This study endeavored to evaluate the vertical effects of non-extraction, anterior mild open bite treatment in adult patients with the Invisalign system. A plethora of evidence lends credence to the idea that skeletal open bite patients tend towards high mandibular plane angles^1^ ^,^ ^17^ ^,^ ^18^ and large lower anterior facial heights (LAFH).^7^ ^,^ ^12^ ^,^ ^17^ ^-^ ^19^ Schudy^20^ claimed the main goal of open bite treatment should be to prevent an increased anterior face height and emphasized that molars should not be extruded during treatment. However, the success of anterior open bite treatment is often gauged by positive maxillary and mandibular incisal overlap, which is usually obtained at the expense of adverse sequela. Fixed appliance therapy has a tendency to worsen the vertical dimension in open bite patients, who more often present as hyperdivergent, long-faced individuals.^6^ ^,^ ^17^ ^,^ ^21^ ^,^ ^22^

Non-surgical options for correction of anterior open bites in adults are limited. It is commonly thought that extractions enable a bite-closing effect by allowing protraction into the extraction space, thereby decreasing the palatomandibular wedge. The literature regarding extraction treatment does not support this idea. In actuality, protraction during space closure may cause occlusal movement of the posterior segments, which essentially cancels out the so-called “wedge effect”.^23^ ^,^ ^24^

Post-treatment increases in lower anterior facial height have been observed in non-extraction treatment as well.^25^ Anterior segments must be extruded via elastics, or posterior segments intruded, to achieve bite closure with fixed appliances. The multiloop edgewise archwire (MEAW) technique employs anterior elastics to achieve positive overbite, to correct the cant of the occlusal planes, and to address the mesial inclination of posterior teeth.^26^ Although the technique has proven capable of attaining bite closure, it is mainly via anterior extrusion.^27^ Previous cephalometric evaluation of the technique revealed insignificant changes in lower anterior face height and in the mandibular plane angle during treatment.^27^ ^,^ ^28^

Bite closure via extrusion of anterior teeth may not be indicated for all adult patients presenting with anterior open bites. Even more, extrusion of the maxillary incisors is deemed unstable.^29^ Some investigators believe that maxillary incisor extrusion in adult patients may compromise the periodontal structures, lead to root resorption and ultimately jeopardize smile esthetics.^10^ Without the use of skeletal anchorage devices, true molar intrusion is very difficult to be achieved in adult patients using fixed appliances.^30^

### Angular measurement changes

In this study, the decision to split the occlusal plane angles was based on the report by Nahoum^7^ that it would be inaccurate to use the same plane in open and deep bite cases and; instead, he defined two separate planes: maxillary (SN-MxOP) and mandibular (SN-MnOP) occlusal planes. SN-MxOP showed a statistically significant mean increase of 2.6^o^. Conversely, Sn-MnOP significantly decreased by a mean of 4.6^o^, indicating again that the bite was closed either by incisor extrusion and/or molar intrusion. MP-MnOP increased significantly by a mean of 4.5^o^; this finding indicates a decreased steepening of the mandibular occlusal plane due to bite closure. A statistically significant mean decrease of 0.9^o^ in the SN-MP angle and mean decrease of 0.9^o^ in the SN-GoGn angle was observed, which we can attribute to counterclockwise rotation of the mandibular plane. For the sake of reliability, two mandibular planes were utilized: 1) one plane connecting inferior gonion to gnathion; 2) one plane connecting inferior gonion to menton. We would expect to see a higher mean value with SN-MP than we would with SN-GoGn, which was the case. However, the mean post-treatment decreases in both planes were equivalent. This rotation was not substantial enough to decrease the MP-MnOP angle, as the treatment effects on the mandibular occlusal plane compensated the skeletal autorotation. SN-PP did not display any significant change, as we might expect in a non-growing patient. 

### Linear measurement changes

**Figure 4:**
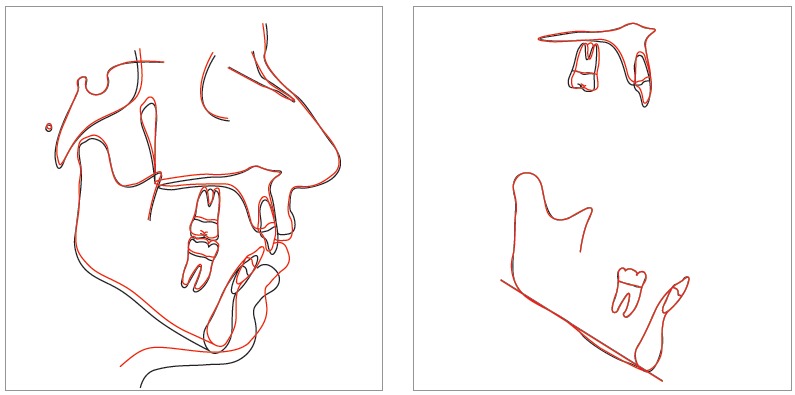
Sample superimposition (patient #12): black = before; red = after.

Following significant mandibular plane closure, there was also a statistically significant 1.5-mm decrease in the lower anterior facial height. Overbite substantially increased, with a statistically significant mean of +3.4 mm; this can be attributed to a combination of molar intrusion and incisor extrusion, as 1 mm of molar intrusion can lead to 3 mm of anterior bite closure.^29^ U1-PP increased by a mean of 0.5 mm and U6-PP decreased by a mean of 0.4 mm. Although this indicates general extrusion of the maxillary incisors and intrusion of the maxillary molars, these changes were not statistically significant. However, L1-MP increased significantly by 0.8 mm and L6-MP decreased significantly by 0.6 mm. This indicates statistically significant mandibular molar intrusion and mandibular incisor extrusion in this sample. These results reveal that, overall, most of the dental treatment effects were greater in the mandibular arch (Fig 4). One possible reason for significant mandibular changes is that interproximal reduction (IPR) was prescribed more in the mandibular arch; this would lead to more mandibular incisor extrusion during retraction and space closure. 

In this study, Invisalign successfully achieved anterior open bite closure via positive incisal overlap without negatively impacting the vertical dimension. The changes observed in the vertical parameters displaying statistical significance are indicative of bite closure and decreases in the vertical dimension.

As previously noted, fixed appliance therapy has the potential to exert unwanted extrusive forces that may enhance the open bite and consequently worsen the vertical dimension. Additionally, anterior-posterior (AP) elastics used with fixed appliances tend to have extrusive effects that increase the vertical dimension. The majority of the present sample presented with an Angle Class I malocclusion, with only six Class II patients. Klein^31^ reported control of the vertical, and even a decrease in the vertical dimension (SN-MP), in his study examining Class II correction with Invisalign and elastics. This finding was attributed to the constant presence of aligner material. The improvements in the vertical dimension seen in this study are akin to those observed subsequent to molar intrusion in skeletal anchorage cases, i.e. reduction in occlusal plane angle, mandibular plane angle, and lower anterior facial height.^10^ ^,^ ^30^

It has been previously postulated that Invisalign exerts a bite-block effect during treatment.^32^ This functional appliance aims to control maxillary vertical skeletal and dental growth by including an acrylic portion in the occlusal region, greater in size than the patient’s normal vertical dimension.^29^ Typically, the amount of acrylic in the first molar area of these bite blocks may range in thickness from 5 to 10 mm; this induces an artificial increase in the vertical dimension, thereby triggering a muscular response that creates a vertical intrusive force in the posterior segments, leading to counterclockwise rotation of the mandible.^33^

It is unlikely that Invisalign has the ability to exert the same intrusive forces in adults as we observe in children with bite block therapy. The thickness of each Invisalign aligner is 0.030 inches^34^ (equivalent to 0.76 mm), which, when combined in both dental arches, may not have adequate thickness to considerably exceed the freeway space, enough to create a neuromuscular response. Additionally, functional contact of opposing teeth occurs approximately 18 minutes per day,^29^ which is not of long enough duration to exert a significant intrusive force.^35^ Intrusive forces from the aligner must be requested by the provider in the ClinCheck^®^ software (Align Technology, Santa Clara, CA, USA) and programmed into the trays through the Invisalign technicians. The combined effect of the sequential progression of trays, with judicious selection and placement of attachments, ultimately dictates the amount of intrusion and tooth movement clinically observed. The advantage of Invisalign in treating open bite malocclusion stems mainly from its full occlusal coverage effect. While intruding dentition, adverse or unwanted reciprocal extrusive movements are less likely to occur because of the presence of the aligner.^15^ ^,^ ^16^

As adult open bite malocclusions are uncommon and their treatment with Invisalign is a relatively new approach, one limitation of this study was obtaining a large sample size. Additionally, the retrospective nature of this study did not enable control of all treatment variables. Each case was treated with a different ClinCheck set-up that was contingent upon the patient’s specific diagnosis as well as the orthodontic provider’s devised treatment plan. Depending on the treatment warranted, providers may have either primarily requested molar intrusion, or anterior extrusion, or a combination of both. Future studies should incorporate a matched control group treated solely with fixed appliance therapy, for further comparison of these modalities’ effects on the vertical dimension. Research focusing on the amount of molar intrusion that can be achieved with the appliance would be of great value. Vertical elastics and IPR may play a role also as for the results achieved in the patients. Lastly, prospective investigation lending insight into relapse of open bite cases treated with Invisalign is vastly important in order to appreciate the appliance’s capacity to preserve control of the vertical. 

## CONCLUSIONS

1) The Invisalign system is a therapeutic modality that can be effectively employed in non-extraction treatment of adult anterior mild open bites. 

2) Bite closure was mainly achieved by a combination of counterclockwise rotation of the mandibular plane, lower molar intrusion and lower incisor extrusion. 
